# Art in transit: Mobility, aesthetics and urban development

**DOI:** 10.1177/00420980221087035

**Published:** 2022-04-21

**Authors:** Theresa Enright

**Affiliations:** University of Toronto, Canada

**Keywords:** aesthetics, infrastructure, mobility, public art, public transport, urban transit

## Abstract

High-profile architecture and design, alongside integrated arts and cultural programming are now ubiquitous features of public transit networks. This article considers how and why transit-based arts and cultural programmes are proliferating globally as well as the impact of these programmes on transit and urban dynamics. Through critically analysing the discourses surrounding different transit art initiatives and the institutional structures which support them, this article shows how transit art is used today for varied – and often contradictory – ends. Based on this, it argues that we should not uncritically celebrate the rise of transit art as an unmitigated civic good. Rather, we must situate the rise of transit art within a political and aesthetic economy in which art has become ‘expedient’, and contend with the way transit art is implicated in elite, exclusionary and unsustainable processes of urbanisation.

## 摘要

如今，引人瞩目的建筑和设计以及综合艺术和文化项目已成为公共交通网络的普遍特征。本文探讨了基于公共交通的艺术和文化项目如何以及为何在全球范围内激增，以及这些项目对公共交通和城市动态的影响。通过批判性地分析不同交通艺术计划的话语和其背后的制度结构，本文展示了交通艺术今天如何被用于不同的—而且往往是相互矛盾的—目的。基于此，我们认为我们不应该不加批判地为交通艺术的兴起而欢呼，将其视为一种不折不扣的公共物品。相反，我们必须将交通艺术的兴起置于艺术已成为“权宜之计”的政治和美学经济背景中，并反对交通艺术所代表的精英化的、排斥性的和不可持续的城市化进程。

## The art of public transport

Art has been a fast-rising trend in public transportation over the past 30 years and investment in the arts broadly construed is now a standard element of new capital projects and upgrades as well as everyday operations. In virtually any large metropolis, public arts initiatives are prominently sited on urban mobility systems, positioned as integral elements of both transit and urban development agendas. A survey of more than 90 cities conducted by the Creative Mobilities policy network in 2018 concluded that the association of arts and cultural policy with transit and mobility is a ‘real trend in all public transport but also private transport… in all regions of the world’ (Marcolin, 2019). Indicative of the spread and significance of this dynamic, in 2019, the International Association of Public Transport (UITP) declared ‘The Art of Public Transport’ the theme of its agenda-setting biennial summit (UITP, 2019).

Iconic station architecture and blue-chip installations are highly visible features of arts in transit programming, but transport agencies are also integrating art, architecture, cultural activities and design (increasingly linked together simply as ‘art’) into a wide variety of initiatives at multiple locations and scales. Examples include track-side graphic arts murals, in-station musical concerts, on-vehicle photography exhibitions, mobile operas and theatrical performances, poetry in motion competitions, interactive planning and visioning exercises, collaborative digital games, and themed tickets and route maps. No longer a decorative afterthought, arts are becoming integrated into station and vehicle design from the outset and included in long-term planning. Transit arts may be permanent or temporary; they may be objects, events or relational processes. They may be low- to no-cost endeavours or they may be billion-dollar legacy investments. They are most commonly associated with metro and commuter rail systems, but are also becoming ubiquitous in bus, bus rapid transit (BRT), light rail and other modal networks.^1^

The pairing of art and transport reflects a promising orientation for advocates who insist on the necessity of revitalising public transit and transit culture in the current conjuncture (see Orol, 2017; Taylor-Hochberg, 2017). Art can valorise public infrastructure, enhance the commuting experience and build common visions of the ‘good’ in urban society. It can also be mobilised for elite and exclusionary forms of transit and city-building that aggravate social and spatial injustices. Transit art, like public transit more generally, is pursued unevenly and for certain purposes and always benefits some while harming others. This tension prompts questions about the cultural industries of transportation, the aesthetics of transit infrastructure, the mobility of cultural policy tied to transit networks, and the spatial politics configuring 21st-century cities. What explains this close imbrication of art and transport? How did art come to be understood as an incontrovertible industry ethos and as an enticing antidote for varied urban ills? And what might a focus on art reveal about the relationship between aesthetics, transport and contemporary urbanisation? Existing scholarship says remarkably little in response to these inquiries and has yet to understand the reasons behind the ubiquity of transit art programmes or their impact and significance.

This paper offers a broad framework that locates transit art as an important site of cultural and creative policy articulation and identifies some critical tensions accompanying this trend. Through an analysis of transit art programmes around the world and the ideological structures that condition them, I show how nebulous notions of art and culture pervade transport discourses to express a diverse range of objectives and desires. Art and culture are politically convenient, legitimating very different sorts of policies and projects while bridging the interests of multiple stakeholders; they are good business, promising broad social and economic returns on investment; and they are enchanting, positioned as an easy panacea for complex and intractable problems that urban and transport authorities have repeatedly failed to solve. Whereas art is treated as a progressive common-sense within the transport sector, the work that art is positioned to do is quite diverse and its impact is uneven.

I make two main claims. First, in the realm of contemporary transport, art has become an ‘expedient’ pretext for ‘sociopolitical amelioration and economic growth’ (Yúdice, 2003: 10), obscuring processes that are more complicated and normatively ambiguous. Second, with the widespread consensus on the instrumental value of art, its critical potential is diminished. As it gains popularity, the art of public transport risks becoming depoliticised; hollowed out of any specific content and unlikely to disrupt the hegemonic neoliberal urban processes in which it is embedded. Through these two dynamics, art risks aestheticising transit politics. That is, art is increasingly being used to stylise policymaking and investment and to defuse and mask conflicts over urban and infrastructural development. Yet these ideological uses are disavowed through the assumption of art’s autonomy and progressiveness. As public transit today is an important modality of urban power and site of struggle and contention, transit art plays a crucial mediating role. It can illuminate political dynamics – of for, example, racism, gentrification, democratic participation and climate action – and the stakes of different transit visions and policies, or it can hide these expressions of power and relegate them to extra-political realms.

This research is based on a qualitative and interpretive examination of the role of arts and arts programming in public transit networks in cities around the world. The analysis draws on planning documents, policies and public statements about transit art programmes in over 60 cities; media reports about prominent transit arts initiatives; 12 semi-formal and informal interviews conducted in 2019 with architects, arts workers (artists, curators, guides, managers and consultants), designers and transit representatives involved in formal art in transit programmes in Brussels, Hong Kong, London, Melbourne, Montreal and Stockholm; and participant observation at the 2019 UITP Summit and on formal and self-guided transit art tours in Lisbon, London, Stockholm and Toronto. I use these various approaches to trace how transit art policies are understood, encoded and enacted by different actors, as well as how they are embedded in urbanisation dynamics.^2^

In what follows I begin by charting the rise of transit art programmes globally from the 1990s onwards, situating this emergence within a context of arts-led urban regeneration schemes. I then employ critical urban theory (Brenner, 2009) and a conflict-attuned approach to public art and urbanisation (Deutsche, 1996; Hall and Robertson, 2001; Miles, 1997) to assess four main contemporary justifications for transit art programming –*image enhancement, creative placemaking, urban regeneration* and *cultural sustainability –* demonstrating how even seemingly innocuous goals are often fraught with political and ideological values which render art a fungible political force. I conclude by reflecting on the role of art in urban mobility transitions, and prospects for further politicising the art of transport.

## A platform for art

Transit architecture, arts and design initiatives (collectively, ‘transit arts’) have long had a profound influence on how, why and where people move through the city; how they relate to the urban environment; and how they understand their relationship with fellow commuters and denizens (Ovenden, 2019; Ström, 1994). In the heyday of civic design in the early 20th century, transit networks were seen to be crucial public infrastructures and arts represented the prestige of their respective polities and cemented values of collective life.^3^ Moscow’s palatial metro stations, for example, were built by Stalin as an ‘immense iconography of power’, symbolising the capacity of the state and manifesting the radiant soviet future (Buck-Morss, 1995: 22). The art and design elements of Paris’ metro similarly embodied modernist dreams of technological and cultural prowess (Ovenden, 2009). In New York and London, the civic function of early transit art was interwoven with customer service imperatives and corporate identities, bridging the ideals of the City Beautiful movement with corporate branding and organisational dynamics (Bloodworth and Ayres, 2006; Bownes et al., 2012; Fitzpatrick, 2009; MTA, 2020). In Buenos Aires, Caracas and Mexico City, the design and architecture of metro systems was explicitly modelled on European counterparts, used to mark Latin American cities as ‘civilised’ and to mould riders into modern urban subjects (Kingsbury, 2017; Ström, 1994).

At the same time, transit art in its early articulations was just as likely to serve prosaic functions as grand ambitions. Historically, one of the most common reasons for including art on transit was to combat the alienating environment of underground rail. Even simple artistic or design adornments, such as changes in station lighting or train colour, could boost ridership and make the commuting experience more appealing (designer, 2019). As Ström (1994) notes, transit arts such as tile colours and built-in monuments were also used to ‘landmark’ significant places, buildings or historical sites, punctuating the repetitive underground circuit and linking to the ‘real’ city above. Furthermore, art was an essential tool of wayfinding. Built-in design and artistic elements were utilised in transit spaces to orient travellers, to move crowds efficiently, and to usher commuters from station entrance to egress (architect, 2019).

However, in many places by the mid 20th century, the exuberance for transit art waned as public priorities shifted away from quality design towards more rational concerns and away from mass transit towards roads and highways. The dominance of functional design intensified into the 1970s where urban financial crises and structural adjustment forced rounds of austerity and burgeoning neoliberal ideologies hollowed out formerly vibrant notions of public space and public services.

At the turn of the 21st century, however, urban authorities, transport professionals and commuting publics began to re-realise the ‘aesthetic potential’ of transit (Ström, 1994: 7). Initially inspired by heritage preservation and culture-led regeneration movements, the use of art to resymbolise and reconfigure ageing transit infrastructure coincided with strategies to position culture at the forefront of post-industrial transformations (Deutsche, 1996; Paddison and Miles, 2020; Sharp et al., 2005). The movement of wealth back to central cities at this time also provided an incentive for municipal authorities to reinvest in public transit amenities to attract highly desired ‘creative class’ urban residents (Florida, 2002). The rise in art in transit programmes coincides with the triangulated urban agendas of cultural regeneration, transit investment and expansion, and the leveraging and creative reworking of infrastructural assets for economic development.^4^ Since the early-1990s, and ramping up in the 2010s, there has been a noticeable turn to rehabilitate architectural and design excellence and to institutionalise arts and cultural programming in transportation networks.

To illustrate how art became more solidly incorporated into urban economic and social development agendas, consider the US Federal Transit Agency (FTA, 1995) circular, which declared that ‘Good design and art can improve the appearance and safety of a facility, give vibrancy to its public spaces and make patrons feel welcome’. These principles and related FTA guidelines and policies have been a major catalyst in securing public funds for transit art and in developing formal public art programmes within transit agencies. Transport Canada (2011: 1–2) has similarly encouraged the widespread adoption of transit art, declaring that art can ‘provide an attractive environment for business and the public; increase public safety; add vibrancy to streets, stations and vehicles; improve passenger travel experience; highlight local culture and heritage; and offer an expression of the city’s and transit agency’s identity’.

Consequently, across North America, there has been a veritable explosion in ‘transit arts’ and ‘art in transit’ programmes.^5^ Transit authorities in Boston, Chicago, Dallas, Los Angeles, Montreal, New York, Philadelphia, Phoenix, Portland, Seattle, St. Louis and Toronto all have dedicated art in transit programmes of one sort or another, while in still more cities, art is incorporated into transit networks on an *ad hoc* basis, tied to select capital projects, administered through city-wide public art programmes, or provided by private developers of transit-oriented neighbourhoods.

There have been parallel patterns of expansion and institutionalisation globally. In Europe, transit agencies in Barcelona, Brussels, Lisbon, Munich, Naples and Warsaw prominently integrated transit art and architecture around the turn of the 21st century. At the same time, more established institutions such as London’s Art on the Underground were revamped, expanding their purview and actively curating high-profile exhibitions and installations (Bownes et al., 2012; Transport for London, 2021). In Asia, Australia, Latin America and the Middle East, the strategy of using transit networks as a platform for prominent architecture, art and design has been replicated in aspiring global cities such as Beijing, Buenos Aires, Delhi, Dubai, Melbourne, Mexico City, Santiago, Sao Paolo, Seoul and Singapore, as well as in less likely places such as Kaohsiung, Medellin, Pyongyang and Taipei. Across Africa, art has long appeared in mass transit systems in informal ways (e.g. the elaborate privately-owned *matatus* of Nairobi), but publicly sanctioned transit art is proliferating and is being seized upon by multiple stakeholders as something to be celebrated and capitalised upon (see, e.g. Akpokiere, 2012; My Citi, 2021).^6^

## Art as a panacea

While transit arts continue to perform many of the same civic, humanising, landmarking, wayfinding, and public relations functions they had throughout the 20th century, as they rise in stature and prevalence, they are being put to an ever-expanding set of uses. Today a wide variety of sometimes-complementary and sometimes-conflicting policy objectives support the inclusion of public art in transit projects. Transit art is figured as a panacea for a host of urban challenges, tasked with everything from increasing public safety and revitalising neighbourhoods, to providing an attractive environment for business and stimulating economic development, to overcoming social discord and restoring democracy, to decreasing greenhouse gas emissions and forestalling planetary apocalypse (see, e.g. Cura, 2001; SubArt, 2017). Is it too much to expect transit art to serve these ever-diversifying functions? Can all of these be achieved in concert?

Delving beneath the gleaming surfaces of transit art and critically examining the claims and contributions advanced by transit art advocates, what Rose (2016) calls the discursive ‘production’ of visual culture, reveals several main justificatory frameworks which are used by politicians, transit operators, policy-makers, developers, community members and aesthetic workers, to frame art in transit policies and their impacts. The four frameworks outlined below are not exhaustive of the many claims raised by transit art practioners and commentators. They represent, rather, the most prominent ensembles of ideas, concepts and narratives – derived inductively – through which the meaning and value of transit art is assigned, articulated, ordered and interpreted. While these frameworks are multivalent, inconsistent and contested, it is evident that as the role of art has expanded in an unprecedented way into the political, social, economic and environmental realms of transport, it is increasingly becoming instrumentalised and viewed primarily through its utilitarian impacts. A central tension runs across and within these discourses: whether art’s contribution to the construction of vibrant and inclusive civic infrastructures which support shared collective life is compatible with its entanglement in market-driven logics which animate competitive and exclusive urbanisation processes.

### Image enhancement

One of the most common reasons that transit authorities give for including art is simply to make the network more pleasant. Speaking about Munich metro’s programme of architectural revitalisation, Rolf Schirmer from the city’s subway planning council argued that transit stations should ‘radiate a positive mood’ (in Hackelsberger, 1997: 11). Mead (2019), Chief Architect for Hong Kong’s Mass Transit Railway (MTR), claims that art can ‘humanise what is often an anonymous and dehumanising space’, and Abdulla Yousef Al Ali, CEO of Dubai’s Roads & Transport Authority’s Rail Agency, boasts that the arts livery on Dubai Metro carriages has effectively transformed the network into a ‘moving icon of happiness’ (Maceda, 2015). Ideally, artistic elements should make passengers’ travel more enjoyable and should be consumed as a fleeting distraction from the otherwise routine drudgery of commuting. With some notable exceptions, transit art, like public art more generally, tends to be what Phillips (1998: 95) calls ‘minimum risk’. In many places, art that is too controversial faces scrutiny (arts worker 2019; architect 2019).

Pleasing and appeasing art is particularly attractive in situations where people have stressful or dangerous commutes or where transit networks and their surrounding urban forms are in disrepair. The arts-forward renovation of Medellin’s metro in the aftermath of civil war was defended on the grounds that art improves the image of the network, thereby promoting safety and social cohesion. Under the banner of social urbanism, Medellin’s transport network was refurbished to connect the divided city and support the mobility needs of working-class residents of the *comunas* (Doyle, 2019; Sotomayor, 2017). Art in transit supported these objectives, while reasserting the value of peaceful coexistence in shared public space and giving a sense of swift spatial transformation. More pointedly, unassailable religious symbols, such as the Virgin Mary were placed in metro stations to prevent targeted attacks by Catholic cartels and militias (99 Percent Invisible, 2018). Image enhancement here goes well beyond surface beautification. Indeed, today, under the banner of Metro Art are included a range of cultural programmes, from exhibitions, to concerts, to libraries and book exchanges which serve a remedial function renewing public goods and public life and distributing resources to the most marginalised.

Medellin presents an extreme example of how art is positioned to enhance positive affects and to repair physical and social divides, but it is hardly an exception. Indeed, across North America and Western Europe, the reinvestments in transit art beginning in the 1980s and 1990s were a response to urban and infrastructural decline (Ström, 1994: 7). Armed with a technocratic view that public art could alleviate the symptoms of pervasive urban crises, municipal and transit authorities approved aesthetic projects to sanitise the image of crumbling public assets. Unlike in Medellin, these relatively cost-effective art programmes would stand in for more systematic upgradings of vital infrastructure and broad social and economic reforms.

For defunded and/or stigmatised transit systems, image-enhancement through transit art is also an opportune way to solve, or more cynically, to suppress urban unrest. In the first wave of the transit art revival, beautification was frequently wedded to crime-prevention through environmental design pradigms. In addition to hostile elements such as blue lights and background classical music which are meant to reduce drug-use and loitering respectively, targeted public art campaigns were deployed to enhance perceptions of public safety and to deter ‘delinquency’, while simultaneously shifting the blame for disrepair onto individuals. With the understanding that empty walls ‘invite vandalism’, sanctioned artworks in Los Angeles, for example, were strategically placed in areas that are prone to graffiti as both a public safety and a cost saving measure (Cura, 2001). Decorated bus stations in Seattle and Portland serve a similar function.

Most infamously, in New York City, the anti-graffiti Clean Train Movement emerged alongside MTA’s official programme of Arts for Transit and Urban Design, both pursued as tactical counterpoints to rehabilitate transit spaces (Austin, 2001). ‘Cleaning up’ the subway was not just about maintenance and heritage preservation, but was simultaneously a key feature of the racist broken windows paradigm that has driven MTA policing policies in recent decades. Nor is this use of arts to endorse penal urban policy restricted to the United States. Stockholm’s world-renown *tunnelbana* art is itself an anti-vandalism measure, supported by a strict municipal zero-tolerance anti-graffiti policy (arts worker, 2019). As train graffiti travelled from New York around the globe, so too did the art and penal policies to prevent it. These revanchist articulations of image-enhancement – adopted to varying degrees depending on the city – are particularly offensive when the very same art forms that were once criminalised (e.g. graffiti) are later appropriated and evidenced of the cultural value of transit institutions.^7^

Creating beautiful transit spaces and cultivating a joyful and safe experience of commuting through enhancing the image of transit is, on its face, rather benign. But it begs the question: safety and joy for whom? And at what cost? Art can be used to modulate environmental and social conditions and can transform broken and banal spaces into repaired and remarkable ones. Yet, when arts programmes are tied to defensive schemes and hierarchical metrics of value, or when art plays a part in the punitive cultivation of a specific set of citizen sensibilities, image enhancement can also gloss over exclusionary social dynamics. Aestheticising transit space through prevailing currencies of race and class can thus segregate as much as it can suture the urban fabric.

### Creative placemaking

The limits of transit art as visual improvement and concerns about the appropriation of marginalised aesthetics are particularly important in projects that aim at creative placemaking. For its advocates, creative placemaking refers to the commissioning of art as a fulcrum for social development and political empowerment (Courage and McKeown, 2018; Courage et al., 2020; Markusen and Gadwa, 2010). Increasingly, creative placemaking is used in infrastructural projects to modulate the visibility of infrastructure, and to enhance community planning and benefits (see, e.g. Kovacs and Biggar, 2018). Transit agencies in particular have been keen to recognise art’s ability animate space and ‘to create meaningful connections among sites, neighbourhoods, and people’ (MTA, 2020). While similar in function to landmarking, placemaking seeks to actively reconfigure meanings, attachments and relations; to ‘transform the landscape and enhance the public dialogue’ (Valley Metro, 2008). Particularly important here is that unlike much public transit art which is merely ‘affixed to the wall’ after the fact (architect 2019; arts worker 2019), creative placemaking promises to intervene in the process of design and construction.

Transportation for America (T4A) (2017: 6) defines creative placemaking:

Creative placemaking harnesses the power of arts and culture to allow for more genuine public engagement – particularly in low-income neighbourhoods, communities of colour and among immigrant populations – in the development of transportation projects… Done right, creative placemaking can lead to both a better process and a better product…

At its most successful, creative placemaking in the transport sector is used to develop a sense of community needs, identities, values and aspirations; to use these in the formation of place-distinctiveness and social ties; and to enhance local feelings of infrastructural ownership through civic participation.

Arts and culture are a tool to address transportation challenges and to build transit systems that more equitably serve communities of colour, low-income people and other disadvantaged groups (T4A, 2017). The use of an arts-based programme of collaborative placemaking in Boston’s Orange Line in the late 1980s, for example, enabled hundreds of artists and residents to ‘reclaim the cultural meaning of their lives’, to rebuild communities threatened by social and political conflict, and thus to empower citizens and commuters (Breitbart and Worden, 1994: 86) From the Transnational Trolley Project in El Paso, Texas, to GoBoston’s Visioning Lab, to Moving Stories initiative in Indianapolis, many projects across the United States are engaging riders and residents in generating transit solutions and organising for mobility justice through the creation and use of public art (T4A, 2017).

Other creative placemaking strategies focus more on representation, outreach, and the articulation of shared identity. Melbourne’s famous ‘art trams’, which have been a conspicuous symbol of the city since 1978, are designed with such an eye to local culture and heritage. Each year, the project invites Victoria-based artists to design graphic arts tram-wrappings. As the vehicles move through the city, they act as highly visible mobile canvases, dialogically interacting with the environments they pass through. These works frequently highlight the city’s Aboriginal history and cultural diversity (Melbourne International Arts Festival, 2018).

There is undoubtedly much to celebrate in these sorts of initiatives (similar practices exist in Cape Town, Delhi, London and Toronto among others). Without minimising the very real positive impacts of democratising engagement and expression, however, some of the more zealous claims of creative placemaking should be seen with suspicion. Public art critics, for example, argue that this sort of placemaking frequently relies on unproblematised and exclusionary notions of place and community (e.g. Deutsche, 1996; Hall and Robertson, 2001; Miles, 1997). Moreover, even when participants might feel empowered by such works, it is rare that participation in arts programmes substantially increases the capacities of residents to determine their own realties, and repossesses self and space against violent machineries of urban development. Art can undoubtedly give residents some influence in processes of transit-oriented urbanisation, but more insidiously, it can also mask lingering conflicts and put a human face (sometimes quite literally) on otherwise overwhelming and dispossessing projects.

Placemaking art is enticing to such a wide range of stakeholders precisely for its ability to manufacture consent among them, alleviating the negative impacts of construction and bridging the sometimes-irreconcilable interests of community and developers. In Melbourne’s Metro Tunnel project, the Creative Programme employed designers, curators and ‘place managers’ not only to reflect community needs, but to explicitly to mediate disputes between stakeholders (Rail Projects Victoria, 2021), exemplifying what Legacy (2016) has called the consensual and post-political turn in Australian transport planning. The targeted use of placemaking art in so-called ‘priority’ low-income and racialised neighbourhoods also raises flags about the conflict between creative initiatives that are intended to promote inclusive neighbourhood development through the arts, and those that are used to deflect and defer spatial conflict and to build consensus. This is the case in Toronto, where community art projects associated with the construction of Toronto’s Crosstown light rail have all been sited in low-income and racialised communities where significant battles persist over the infrastructure development, its distribution of benefits, and its potential for displacement (see, e.g. Rankin and McLean, 2015).

The more nefarious manipulations of community, identity and place through the arts come into even sharper focus when creative placemaking is situated within broader programmes of urban regeneration (Blokland, 2009; Burns and Berbary, 2021; Fincher et al., 2016). Aesthetic disciplines are at the forefront of re-ordering post-industrial space, and in this shift, placemaking is increasingly being used to justify elite property-led forms of city-building. With respect to art in transit, creative endeavours may be acclaimed in the intangible terms of social development, but the bottom line is more often defined by crude market calculations. Arts interventions generate fast economic returns and in resymbolising transit spaces as productive assets, they can thus ‘help secure consent to redevelopment and to the restructuring that make up the historical form of late capitalist urbanism’ (Deutsche, 1996: 10). The beautification of transit thus cannot be divorced from multiscalar projects of gentrification which ‘upgrade’ neighbourhoods materially and aesthetically, while displacing the most marginalised residents (Bridge, 2006; Mathews, 2010; Summers, 2020).

Today, the creation of site-specific public art is a valuable marketing opportunity for both public transit agencies and private developers as relatively modest investments have huge returns in terms of public relations and increases in land and real estate values. As Rosenfeld (2012) writes of the privately sponsored ‘community’ murals at Wilshire Vermont Station in Los Angeles, pairing transit-oriented development with public art is profitable business. ‘The private marketing benefits, in real-dollar terms, of this modest public art investment are almost inestimably high…Dollar for dollar, investments in public art may provide the highest financial returns of any funds committed to an aspect of a transit project’.

Placemaking not only contributes charismatic atmosphere, but in differentiating niche spatial products, it plays a role in attracting investments, contributing to tourism, catalysing surrounding developments, adding to land values, and closing rent gaps. Increasingly, architects, artists and ‘cultural placemaking’ professionals utilise participatory site-specific methods to gain the legitimacy of place-based works, while working within a globalised marketplace of infrastructure and urbanism (consultant, 2019; designer, 2019; on this trend of serialisation and specificity in placemaking more generally, see Kwon, 2004). Not only are different kinds of place-based practices glossed over in transit art narratives, but the praise for these projects often obscures the commodification dynamics that also inhere in their making.

### Urban regeneration

Beyond individual nodes, stations and neighbourhoods, aesthetic upgrades of transit have transformative potential for the transit network and for the city at large. Naples’ lavish ‘art subway’, exemplifies the growth-optimising cultural regeneration programme of the aspiring global city. Despite chronic fiscal shortfalls and severe public utility gaps, since 2003, the city of Naples it has invested in a comprehensive programme to transform the underground transit system into a world-renown museum on the premise that this will catalyse broader urban change. As the former Mayor of Naples, Luigi de Magistris, reported to *The New York Times* in 2013, the art project seeks not merely transformation of individual metro stations as such, but ‘redefining the spaces of our city’ with a state-of-the-art transit network acting as a metonym for competitive world-class status (Povoledo, 2013).

In Naples, the upscaling of transit through public art was pursued alongside the promotion of other cultural industries, festivals and institutions. This strategy has the benefit of allowing transit authorities to link into existing resources, while also raising the profile of the arts more generally. The Wehrhahn metro line in Düsseldorf, which opened in 2016, also adopts this synergetic approach. Collaboratively designed by artists, architects and engineers to feature a coherent identity, the line was promoted by the city’s cultural department as way to advertise the Kunstakademie Düsseldorf (Arts Academy) and as means to position Düsseldorf globally as a high-ranking node of artistic production on par with Berlin (Dunmall, 2016). San Francisco’s BART Art Masterplan ‘envisions BART as the Bay Area’s cultural corridor…that will help rebrand BART as a world-class, attractive and durable system’ (BART, 2019).

The Arts Programme of London’s Elizabeth Line (formerly known as Crossrail) represents a hyperbolic form of this dynamic. Seven central-city stations, designated the ‘Culture Line’, feature fully integrated specific artworks by internationally renowned artists such as Spencer Finch, Richard Wright and Yayoi Kusama. Each station along the Culture Line has also paired with a notable London Gallery and the design teams are a veritable who’s who of the global architectural and design world. Private corporate sponsors such as, Goldman Sachs, Derwent and Canary Wharf Group, have welcomed the opportunity to share in the ambition and prestige of the project and to partake in the ‘once in a generation opportunity to associate their brand with an iconic piece of London infrastructure and some of the world’s most famed galleries and artists’ (McDougall, 2014). Through its station architecture, public realm and oversight developments, neighbourhood improvements and landscaping, creative partnerships, and real-estate collaborations, the transit arts of the Elizabeth Line are ‘not strictly transport related’ (Pearman, 2016: 23). Indeed, paramount among the objectives of the Elizabeth Line – to which art is integral – are the land value increases of upwards of 30% that the network will unlock around each station (Phillips and Southern, 2019).

The architecture, art and design of the Elizabeth Line are stunning. The station designs notably reference local histories and material cultures, and the integrated artworks of the Culture Line make visible new dimensions of transit space, from the deep time of earth and clouds to the everyday rhythms of mobility and conviviality (designer, 2019; arts worker 2019). Transit arts such as these can offer new perspectives on infrastructure and the relations that comprise it. And yet, art which makes-place can also dis-place.

It is not coincidental that the Arts Programme is sponsored by the same financial and urban development corporations that have undermined public goods, accelerated land privatisation, and driven hypergentrification within London. The civic ambitions of transit art – to democratise culture, to dignify collective goods and to promote social connection (Crossrail, 2021) – are a tragically ironic fit for the prestige projects of what Atkinson (2020) calls the ‘Alpha City’. Surely, the arts associated with the Elizabeth Line cannot be reduced to these corporate functions (indeed, many of the artworks have a critical edge and ‘prestige’ works are accompanied by many lower-profile works and cultural outreach activities), however, neither can the corporate-sponsored Art Programme be understood outside of the colonisation of London and its cityscapes by the interests of finance. Here, profitable corporations accumulate cultural capital and moral standing through the sponsorship of art that is meant to address the problems they have undeniably created. Not only does public art fail to ameliorate the social and political tensions it seeks to address, but it can also be complicit in the obfuscation of the structural roots of inequality.

As artists, designers and architects are increasingly employed to establish the basis for future infrastructural returns, and aesthetics are at the forefront of global gentrification trends, it is worth asking in whose image the city is being built as well as the social and economic consequences of such transformations.

### Cultural sustainability

Finally, the dynamics of image-enhancement, creative placemaking and urban regeneration should be understood alongside claims about transit art’s role in achieving ‘cultural sustainability’ (Soini and Birkeland, 2014; Soini and Dessein, 2016). With culture positioned as a fourth element of sustainability (UCLG, 2010), transit arts are often folded into established ‘green’ development agendas. Indeed, the foremost rationale for investing in arts today is to raise the profile of transit so as to reduce the private automobile modal share of urban mobility and thus to mitigate climate change. ‘As transportation authorities seek new and innovative approaches to getting people out of their cars and into transit, the recognition of the need to create a more inviting public transit environment has led to including public art in transit projects’ (Transport Canada, 2011: 2). Activists, engineers, cultural producers and policy-makers agree that high-quality and attractive transit spaces, as well as habits and cultures of transit, are needed to meet ambitious future visions of zero-emissions shared mobility. However, how they understand the links between art, mobility and sustainability is quite diverse.

It is unsurprising that cities which identify as ‘green’ have also integrated sustainability and arts initiatives. Singapore’s Art in Transit programme, which features artworks by the country’s leading artists on the Mass Rapid Transit (MRT) network, regularly invokes nature, ecology, and the relationship between urbanisation and the natural environment. Art is meant to highlight local places and communities while simultaneously encouraging non-automobile travel and urban sustainability (Land Transport Authority, 2021). This is an example of what Soini and Birkeland (2014: 218) call an arts-based storyline of ‘eco-cultural resilience’.

What is more remarkable is the way transit and arts investments have also been put to use to secure cultures of sustainable transport where they are absent. Transit art is flourishing today in the Gulf states where car-dependent urbanisms, low-density development, and ideologies of automobility prevail. Cities in the UAE, Saudi Arabia and Qatar have recently redirected petroleum profits in pursuit of decarbonised energy transitions premised on the idea that world class cities need world class public transport infrastructure (Furlan and Sipe, 2017; Hannawi et al., 2019). Transit arts here serve a dual function, rebranding externally, while simultaneously transforming local public opinion and behaviour.

The architecture and design firm Perkins +Will (2021) describe the overarching design challenge of the Riyadh metro as how to ‘help convince a car-focussed population, in a city that had no previous experience with public transit, to get out of their cars and onto the train?’ To lure car-drivers, they focussed on elegant and high-quality materials and components to create ‘interest in the new system’. Doha’s metro, constructed in advance of the 2022 World Cup, similarly has luxury architectural and arts initiatives interwoven with customer service and corporate sustainability goals. And taking this pattern to the spectacular extreme, Dubai’s arts programmes comprise flagship stations, themed interior design schemes, train art liveries, large outdoor ‘street’ murals, and an announcement in 2014 by Sheikh Mohammed bin Rashid Al Maktoum, to turn six central metro stations into a set of themed art museums.

Arts initiatives are integral to procuring a luxury travel experience so desired by greening economies. Based on the evidence that ‘image’ plays a significant role in modal preference (e.g. Hensher and Mulley, 2015), high quality and recognisable design, as well as integrated art elements increase the perceived benefits of superior comfort, security and reliability that stand to attract moreby-choice riders. As such, the explicit use of design to lure ridership (Hess and Bitterman, 2008), improve brand recognition (Zigmund, 2021), leverage cultural capital (Enright, 2016) and promote ‘transit culture’ (Jaffe, 2014) has become a common practice.

In addition to public and transport authorities and corporate design and placemaking firms, policy networks like Creative Mobilities and United Cities Local Governments (UCLG), individual transit art specialists, charities like Julie’s Bicycle, and corporate consultancies such as the Yellow Design Foundation, are keen to capitalise on this frontier, marketing ‘strategic synergies between arts, culture and mobility…for sustainable development’ to cities around the world (Creative Mobilities, 2020). These emerging experts are essential for how norms are being produced, who is participating, how cities describe own initiatives and how arts initiatives are mobilised from city to city.

It is undeniable that public transit today must be prioritised to meet the grave and present challenges of climate crises. And creating cultures of decarbonised mobility, including through critical ‘climate art’ (e.g. Miles, 2010), is absolutely needed as part of Green New Deals and energy transitions. However, when arts and culture are packaged as a set of endlessly replicable, adoptable and transmutable ‘fast policy’ products (Peck and Theodore, 2015) and are presented as a means to ‘develop uncomplicated solutions to complex problems’ (Yellow Design Foundation, 2020) they should be viewed with scepticism. Agendas of cultural sustainability, for example, are often articulated in the same breath as ecologically damaging and socially divisive forms of urban development (e.g. Doha’s staggering urbanisation in advance of the 2020 World Cup, or Foster+ Partner’s withdrawal from the Architect’s Declare pledge because of their commitment to designing airports). Sustainability discourses of transit art which rest on flat notions of beauty, innovation and prestige are unlikely to adequately confront the economic and social relations underpinning environmental destruction.

## The limits of transit culture

The growth in popularity of transit art globally, its imbrication in transnational policy networks, and uptake as a best practice of urbanism and urban development signals interest in the transformative potential and political power of aesthetic practices, with art frequently used as an uncritical signifier of positivity, progressivity and profitability. Yet even a cursory look at transit art programmes reveals that they are not all rooted in shared civic aspirations, and they do much work beyond enacting good design for universal betterment. Transit art carries out a whole range of functions that are progressive and regressive, sometimes simultaneously so.

In this article I outline a theoretical and critical understanding of art in transit by analysing key justificatory discourses. In unpacking how transit art is positioned in frameworks of image enhancement, creative placemaking, urban regeneration and cultural sustainability, I claim that even though transit art has largely been advanced on the assumptions that ‘art’ is an abstract force that acts in consistent and predictable ways in pursuit of public good, this is not always borne out in practice. Such a perspective reveals tensions in the work that transit art is positioned to do. Not only are transit art schemes worldwide embedded within powerful spatial processes of capitalism that continuously work to undermine alternatives, but they are also neutralised through professional bureaucratic apparatuses of public art and urban solutionism. Although art can promote active and critical deliberation about public transit, its social and political composition, and its role in the hierarchical production of space, it can also work to uphold existing regimes of transit value, authority and urbanism and ‘to smooth, rather than disrupt, social relations in the city’ (Hall, 2007: 1383). Despite and because of its wide appeal, transit art today risks furthering the latter to the detriment of the former. Transit art programmes are marked by deep ambiguity, and to understand the stakes and impact of transit art, it is necessary to further unpack how art *and* transit are always already embedded in the contested dynamics of urbanisation.

Thus, it also indicates several directions for future research. First, the variety of discourses supporting transit art and its divergent outcomes suggest that contextual factors are essential to how this global trend is being taken up, translated and implemented. In addition to the general patterns outlined here, it is imperative to trace how different financial, institutional and political environments are shaping public art in transit. Second, as transit art becomes more and more accepted as an expedient ‘fix’, the substance of transit artworks and cultural programming becomes, paradoxically, largely irrelevant. Noticeably absent from nearly all conversations about the importance of transit art today are reflections of artists, the contents of artworks, and the kinds of art that may be more or less conducive to achieving the ends to which they aim. More localised and nuanced research on these compositional and consumption dynamics – including explicit attention to public artworks that politicise mobility – would help to fill in the broad strokes analysis presented here. Third, this research suggests that critical transport studies could be enhanced by greater attention to the aesthetic, imaginary, symbolic and cultural dimensions of transportation networks and transit-oriented development. This might include, for example, analyses of how art in transit has engendered gentrification and non-housing related forms of exclusionary development. Fourth, whereas creative placemaking is becoming more commonplace as a scholarly concept (see Courage et al., 2021), this literature has yet to fully engage art in transit practices. More systematically attending to creative placemaking on and around mass transit may reveal the longer term effects of (participative) initiatives of transit arts as well as the non-material effects of public art on the commuting experience. The art in transit phenomenon also invokes more ‘mobile’ dimensions of place and publicity (see Sheller, 2014) which might usefully extend theories of placemaking. And fifth, it is clear that for effective and inclusive energy and mobility transitions, cultural and social transformations must accompany technological and political change. Whereas art in transit is a promising site to lead this transition, the contemporary expediency of transit art and culture suggest that current iterations may not be up to the task. More critical work, both artistic and scholarly, could engage and transform the relationships between art, mobility and environmental justice.

Further attending to the ideologies behind transit art initiatives, developing better ways of framing and evaluating the holistic impact of diverse arts in transit programmes, questioning the organisational and financial conditions of the transport and arts industries that underwrite these, and embedding transit arts within contentious processes of urbanisation can help to point the trend towards the politicisation of transit aesthetics rather than the aestheticisation of transit politics.

## Notes

1. While the focus here is on ‘official’ publicly-supported arts initiatives, unsanctioned arts practices on transit are also flourishing.

2. In sorting through this material, I employed a critical discourse analysis attending to the content of the texts, the social context in which they arise, and the work they are made to do. Attention is paid to the function of these discourses within capitalist relations.

3. This is particularly true for metro (subway) systems, which, due to their size, scale, and impact on the built environment, have a rich legacy of architecture, arts and design. Due to this history, I focus here on metro systems, but transit arts are also important, and increasingly so, to other public transport modes.

4. Art in transit programmes have much in common, for example, with initiatives such as the High Line in New York which re-imagine infrastructural spaces within highly-stylised agendas of post-industrial urbanism (see, e.g. Lindner and Rosa, 2017).

5. The names of these programmes varies by context.

6. Metro systems are relatively rare on the African continent, but publicly-sponsored arts programming is used on commuter rail lines, buses and in emerging BRT networks.

7. The ‘graffiti aesthetic’, for example is now widespread, even in commissioned transit artworks. Dance forms such as Showtime with their origins in urban mobility also move between illegality and distinction. These dynamics of cultural production and regulation are often highly racialised, with criminalised Black cultural forms appropriated by transit agencies as marketable amenities.
